# Dermatologically Tested Apple‐Derived Extracellular Vesicles: Safety, Anti‐Aging, and Soothing Benefits for Skin Health

**DOI:** 10.1111/jocd.70254

**Published:** 2025-07-21

**Authors:** Lucia Sileo, Maria Pia Cavaleri, Luca Lovatti, Giuseppe Pezzotti, Letizia Ferroni, Barbara Zavan

**Affiliations:** ^1^ Medical Science Department University of Ferrara Ferrara Italy; ^2^ Biomedical Research Center Kansai Medical University Osaka Japan; ^3^ Maria Cecilia Hospital GVM Care and Research Ravenna Italy

**Keywords:** biological targets, computational methods, nanoparticles, natural products, pharmaceutical formulations, pharmaceutical processes, pharmacodynamics, pharmacogenomics and pharmacogenetics, pharmacokinetics

## Abstract

Extracellular vesicles (EVs) derived from plant sources, particularly apple‐derived vesicles (ADVs), have gained interest for their biocompatibility and bioactive properties in cosmetic, dermatological, and regenerative medicine applications. This study aimed to evaluate the safety and efficacy of a 2% ADV‐based formulation, assessing its potential for topical use through a series of standardized in vitro tests.

**Materials and Methods:**

The ADV‐based formulation was subjected to genotoxicity (Ames test), corneal toxicity, skin irritation, corrosion, and sensitization assays, following OECD guidelines. Additionally, its anti‐inflammatory and anti‐aging effects were assessed through skin redness reduction and wrinkle parameter analysis over 60 days.

**Results:**

ADVs exhibited no genotoxic, cytotoxic, corrosive, or sensitizing effects, confirming their safety profile. The formulation significantly reduced skin redness (*p* < 0.05) and improved wrinkle length, volume, and roughness over time.

**Conclusion:**

These findings establish ADVs as safe and effective bioactive ingredients, supporting their potential integration into cosmetic and regenerative applications for skin health and anti‐aging treatments.

## 1 Introduction

Extracellular vesicles (EVs) represent a heterogeneous population of particles naturally released by cells into their surrounding environment. These vesicles are integral to intercellular communication, serving as carriers of bioactive molecules such as proteins, lipids, and nucleic acids [1]. Through their cargo, EVs facilitate the transfer of signals and regulatory factors between cells, influencing a variety of biological processes, including immune responses, tissue repair, and cellular homeostasis. The ability of EVs to mediate these processes makes them a subject of growing interest across multiple scientific disciplines [2].

While EVs were initially studied in the context of animal and human cells, the exploration of plant‐derived extracellular vesicles (PDEVs) has expanded significantly in recent years. PDEVs are naturally secreted by plants and share many functional characteristics with their animal‐derived counterparts. However, they also exhibit unique features, including distinct lipid compositions and plant‐specific biomolecules, which contribute to their stability and functionality [3]. PDEVs have garnered attention due to their biocompatibility, low immunogenicity, and ease of extraction from readily available plant materials. In addition to their inherent biological properties, PDEVs are recognized for their potential applications in biotechnology, medicine, and cosmetics. Their natural origin aligns with the increasing demand for sustainable and eco‐friendly solutions, while their stability and scalability make them an attractive resource for large‐scale production [4, 5, 6]. These attributes position PDEVs as a promising tool for advancing therapeutic delivery systems, regenerative medicine, and the development of bioactive ingredients for personal care products. The growing interest in PDEVs underscores their transformative potential in both scientific research and commercial applications. Among plant sources, apples ( *Malus domestica* ) are particularly attractive for their high yield of vesicles and their rich content of bioactive compounds such as polyphenols, pectins, and antioxidants [7, 8, 9]. These components are widely recognized for their health benefits, including anti‐inflammatory, antioxidant, and regenerative properties, making apple‐derived vesicles an innovative candidate for cosmetic applications.

Our prior studies have demonstrated that apple‐derived vesicles exhibit significant potential in promoting skin health. Apple‐derived exosomes, or plant extracellular vesicles, are nanometer‐sized, membrane‐bound structures secreted by apple cells that serve as carriers of functional biomolecules. The work conducted by Martini et al. [10] and colleagues has provided valuable insight into the molecular composition and bioactivity of these vesicles, particularly those isolated from the *Malus domestica* Golden Delicious variety. These vesicles contain a lipid bilayer membrane composed primarily of plant‐derived phosphatidylcholine, phosphatidylethanolamine, and glycolipids, which confer stability in extracellular environments and facilitate fusion with mammalian cell membranes. Encapsulated within the vesicles is a rich cargo of RNA species, including both messenger RNAs (mRNAs) and microRNAs (miRNAs), among which miR‐146a is notably enriched. This specific miRNA has been shown to regulate immune responses by targeting key signaling intermediates such as IRAK1 and TRAF6, which are involved in the Toll‐like receptor and NF‐κB inflammatory pathways. In addition to RNA, these exosomes carry plant‐derived proteins, including enzymatic antioxidants such as superoxide dismutase (SOD), peroxidases, and catalases, which contribute to oxidative stress modulation. They may also include metabolic enzymes involved in sugar metabolism and stress adaptation. Furthermore, metabolomic profiling suggests the presence of phytochemicals such as polyphenols, flavonoids (e.g., quercetin, catechin), and phenolic acids, either enclosed within the vesicles or associated with their membranes. These bioactive compounds are known for their roles in redox balance, antimicrobial activity, and anti‐inflammatory signaling. Functionally, apple‐derived exosomes demonstrate immunomodulatory, anti‐inflammatory, and regenerative properties. In vitro experiments have shown that treatment of human macrophages with apple exosomes leads to a significant downregulation of pro‐inflammatory cytokines such as TNF‐α, IL‐6, and IL‐1β upon LPS stimulation, suggesting that these vesicles may reprogram macrophages toward a less reactive, possibly M2‐like phenotype. This immunosuppressive effect is partially mediated by miR‐146a, which interferes with the downstream signaling cascades of pattern recognition receptors. Furthermore, apple‐derived exosomes are able to enter human cells via endocytosis, deliver their cargo, and exert post‐transcriptional regulation, modulating gene expression profiles in recipient cells. In terms of therapeutic potential, these exosomes exhibit high biocompatibility, non‐toxicity, and stability in gastrointestinal and cutaneous environments, making them suitable for oral, topical, or even injectable delivery systems. Our group's studies also highlight the possibility of integrating apple exosomes into nutraceutical formulations, cosmeceutical creams, and microbiota‐modulating supplements, with emerging evidence suggesting their ability to support epithelial regeneration, improve gut barrier function, and modulate host–microbe interactions. Their plant origin ensures low immunogenicity and allows for sustainable, large‐scale production, positioning them as a natural and innovative tool in personalized medicine and functional food development. Such properties align with the growing demand for natural, sustainable, and effective ingredients in the cosmetic industry. However, despite their promising bioactivities, the safety profile of apple‐derived vesicles remains largely unexplored [11, 12].

The cosmetic industry is increasingly driven by the demand for safe, natural, and multifunctional ingredients that align with sustainability principles. Apple‐derived vesicles offer a unique combination of efficacy and natural origin, positioning them as a potential breakthrough in cosmetic formulations. By establishing a thorough safety profile, this study seeks to pave the way for the integration of apple‐derived vesicles into commercial skincare products, ensuring consumer confidence and regulatory compliance [13, 14, 15]. To address this gap, we aim to evaluate the safety of apple‐derived vesicles through a comprehensive battery of standard toxicity and compatibility tests. These include the Ames test for mutagenicity, corneal and skin irritation assays, corrosion evaluations, sensitization studies, and their impact on skin microbiota. The Ames test is essential to determine the mutagenic potential of these vesicles, ensuring they do not pose genetic risks. Corneal and skin irritation tests assess the vesicles' potential to cause adverse reactions when applied topically, while corrosion tests evaluate their ability to damage tissues. Sensitization studies are critical for identifying any allergenic potential, ensuring long‐term safety for users. Finally, investigating the impact on skin microbiota is of particular importance, as maintaining a balanced microbiome is a key factor in skin health and integrity.

## 2 Materials and Methods

### 2.1 Apple Derived Vescicles

Apple‐derived vesicles (ADVs) used in Zevex (CinnaPharm Italy) were commercially sourced and acquired through a licensed supplier. The vesicles underwent quality control to confirm purity, stability, and bioactivity before formulation. The composition of the cream tested water, coconut kernel, coco‐caprylate, propanediol, ADVs 5% of E^8^, potassium acetyl phosphate, diethylhexyl carbonate, arachidoyl alcohol, glycerin, *simmondsia chinensis* seed oil, caprylic triglycerides, phenoxyethanol, gluconolactone, biphenyl alcohol, leuconostoc root ferment filtrate, lactic acid, arachidyl glucoside, tocopherol acetate, *cocos nucifera* oil, cetearyl alcohol, sodium gluconate, levulinic acid, prunus amygdalus dulcis oil, *Ceratonia siliqua* gum, sodium levulinate, sodium benzoate, ethylhexy, glycerin, tocopherol, citric acid, tartaric acid, gluconic acid.

### 2.2 Ames Assay

The experimental procedures are based on the OECD Guideline for testing of chemicals no. 471 OECD Guideline for the testing of chemicals no. 471 [16] “Bacterial Reverse Mutation Test for the evaluation of the mutagenic potential (Ames assay)”. The bacterial cells, in growth phase, are exposed to different concentrations of the AdVs and mutagenic activity is determined by the capacity of the test substance to induce a significant increase in the number of reverted colonies (histidine‐indipendent mutant, His+ or tryptophan‐independent Trp+) in comparison to the spontaneous reversions occurring in the control cultures. Some chemical agents are not directly mutagen but become so following transformation and metabolic activation occurring in the organism by liver enzyme activity. In order to study this mutagenic effect, rat liver microsomial fraction (S9) has been added. S9 employ admits to identify indirect mutagen substances. According to the Guideline, the number of colonies grown are counted and compared with a negative (untreated) and a positive control (treatment with a mutagenic compound). Bacterial strains: *S. typhimurium* TA 1535, TA 100, TA98, TA 1537 (Sigma, US). These four *S. typhimurium* strains have GC base pairs at the primary reversion site and it is known that they may not detect certain oxidizing mutagens, cross‐ linking agents and hydrazines. Each tester strains contains a different type of mutation in the histidine operon (TA 1535, TA 100, TA98, TA 1537) or in the tryptophan operon (EC WP2 trp uvrA). TA 1535 and TA 100 strains are specific testers for mutagens causing base substitutions. Cross‐linking mutagens may be detected by the DNA repair‐ proficient strain of EC WP2 trp uvrA. The sensitivity of TA 100 is greatly enhanced by the introduction of an R factor, pKM101, which confers ampicillin resistance. The frameshift tester strains used are TA 1537 and TA 98. TA 98, like TA 100, is ampicillin resistant. All *S. typhimurium* strains carry, along with the defect in the histidine gene (His‐), a deep rough (rfa) character, a mutation that causes partial loss of the lipopolysaccharide barrier that coats the surface of the bacteria and increase permeability to large molecules. At the end in these strains, there is a delection of a gene coding for the DNA excision repair system (uvrB‐), resulting in greatly increased sensitivity in detecting many mutagens. For technical reasons, the deletion excising the uvrB gene extends through the bio gene and, as a consequence, these bacteria also require biotin for growth.

Experimental procedure: Ames test was performed on ADV (Zevex Cinnapharm, Italy) sample dilutions with strains TA98, TA100, TA1535, TA1537 of *Salmonella typhimurium* and EC WP2 trp uvrA with and without metabolic activation S9 (Moltox, USA). The samples were analyzed in a concentration range from 5% to 0.05% mg/plate. Water was used as a negative control. Positive controls were chosen according to the Guideline (2‐amino‐anthracene CAS no. 613‐13‐8; sodium azide CAS no. 26628‐22‐8; Daunomycin; ICR191 Acridine CAS no. 17070‐45‐0; Methyl Methanesulfonate).

### 2.3 Eye Hazard Potential

The test follows the OECD 492 guideline and uses a reconstructed human corneal epithelium (RHC) model (Episkin, SkinEthic, Corneal Epithelium) [17] that mimics the structural and functional features of the human cornea. This approach evaluates cytotoxicity by analyzing the damage caused by the test substance penetrating the corneal epithelium and helps assess potential eye hazards. Cell viability is measured through the reduction of MTT [3‐(4,5‐Dimethylthiazol‐2‐yl)‐2,5‐diphenyltetrazolium bromide] to blue formazan, which is quantified after tissue extraction. Chemicals are classified as hazardous if they significantly reduce cell viability below established thresholds.

Upon arrival, plates are handled in a sterile environment, and tissues are transferred to 12‐well plates containing SkinEthic Maintenance Medium. Cultures are incubated overnight at 37°C with 5% CO_2_ and high humidity. The test substance's potential to interfere with MTT reduction is also evaluated by incubating it with MTT solution under controlled conditions. If a blue or purple color develops, additional controls are implemented. Results are expressed as a percentage of cell viability compared to the negative control and calculated using established formulas.

The assay was considered valid if the following criteria were met:

Negative control: mean OD value > 1 and ≤ 2, 5; difference of viability between replicates ≤ 20%.

Positive control: mean viability (expressed as percentage of the negative control) ≤ 50% at 5 min.

Exposure (neat); > 50% at 16 and 120 min (diluted); difference of viability between replicates ≤ 20%.

Test item: difference of viability between replicates ≤ 20%.

After subtracting blanks and applying corrections for background controls, the mean values, standard deviations, and mean relative viability percentages (compared to the negative control) were calculated. Threshold values for the test endpoint are outlined in a prediction model, which is used to determine and classify the ocular hazard potential of test chemicals based on the UN GHS classification system, as detailed:

No Category: mean relative viability > 50% within all‐time treatments.

Category 1: mean relative viability ≤ 50% within all‐time treatments.

Category 2: any other values (other than No Cat or Cat 1).

For coloring test items, Non Specific Color (NSCliving) relative to the D‐PBS Control is evaluated as follows:

NSC_living_ = 100 × (ODtest item (not incubated with MTT)/O.D.D‐PBS (incubated with MTT)).

If the NSC_living_ ≤ 5% only blank subtraction is carried out.

If 5% < NSC^living^ ≤ 50%, blank and appropriate background subtraction is carried out.

If NSC_living_ > 50% the test item is not suitable for this test method.

### 2.4 Corrosion

The objective of the study was to determine whether the test substance causes skin corrosion, defined as permanent damage to the skin, including visible necrosis extending through the epidermis into the dermis. This evaluation was conducted using a reconstructed human epidermis (RhE) model, a commercially available system that mimics the biochemical and physiological characteristics of the skin's outermost layers. The study adhered to the OECD Guideline No. 431 [18], “In Vitro Skin Corrosion: Reconstructed Human Epidermis Test Method.” The method is based on the concept that corrosive chemicals can penetrate the stratum corneum by erosion or diffusion, leading to cytotoxic effects in the underlying cell layers. Cell viability is assessed by measuring the reduction of MTT dye [3‐(4,5‐Dimethylthiazol‐2‐yl)‐2,5‐diphenyltetrazolium bromide, Thiazolyl blue; CAS No. 298‐93‐1] into a blue formazan product, which is quantified after extraction from the tissue. Chemicals are classified as corrosive if they reduce cell viability below the threshold values established by the guideline.

The validity of the assay was confirmed if the following criteria were satisfied:

Negative control (no corrosive): Mean OD value between 0.8 and 3;

Positive control (corrosive): Mean OD < 0.8.

### 2.5 Irritation

The potential for skin irritation caused by the test substance, AdVs (Cinnapharm, Italy), was assessed through an in vitro experiment using a reconstructed human epidermis (RhE) model (Episkin, SkinEthic RHE (RHE/S/17)). The study adhered to the procedures specified in OECD Guideline No. 439 for chemical testing [19]. Both the test substance and the controls were evaluated for their ability to affect cell viability after a 42 ± 1 min exposure period, followed by a recovery phase of 42 ± 1 h. The primary endpoint was cell viability, measured through a colorimetric MTT assay. The results were expressed as percentages of cell viability relative to the negative control.

The assay was considered valid if the following criteria were met:

Blank control: mean OD value < 0.1.

Negative control: mean OD value ≥ 0.8 and ≤ 3; SD of % viability ≤ 18.

Positive control: mean viability (expressed as percentage of the negative control) ≤ 40%; SD of % viability ≤ 18.

Test item: SD of % viability ≤ 18.

Cut‐off values for the endpoint of the test are established as follows:

Irritant (US GHS Category 2): Mean relative viability ≤ 50.

Not irritant (US GHS No Category): Mean relative viability > 50.

### 2.6 Sensitization

The study was conducted to evaluate the in vitro skin sensitization potential [20] of the test substance through the activation of human monocytic cells, serving as a model for dendritic cell response. The assay utilized THP‐1 cells (ATCC TIB‐202), a well‐characterized human monocytic cell line, to assess immune activation by quantifying the upregulation of key surface proteins associated with dendritic cell maturation. Specifically, the expression levels of CD86 and CD54, two co‐stimulatory molecules involved in antigen presentation and immune signaling, were measured following exposure to the test substance. To determine the activation status of the cells, THP‐1 cultures were incubated with the test compound for 24 h, after which fluorescently labeled antibodies targeting CD86 and CD54 were used for detection. Flow cytometry analysis was performed to quantify the fluorescence intensity of these markers, providing an indication of the test substance's sensitizing potential. A substance was classified as a potential sensitizer if the Relative Fluorescence Intensity (RFI) exceeded the threshold values established by regulatory guidelines.

The THP‐1 cells were maintained in RPMI‐1640 culture medium supplemented with 10% fetal bovine serum (FBS), 0.05 mM 2‐mercaptoethanol, 100 U/mL penicillin, and 100 μg/mL streptomycin, under standard culture conditions of 5% CO_2_ at 37°C in a humidified incubator. Prior to flow cytometric analysis, cell viability was assessed using propidium iodide (PI) staining, ensuring that observed changes in marker expression were not due to cytotoxic effects. Following exposure to the positive control, negative control, and test compound, the cells were resuspended in fluorescence‐activated cell sorting (FACS) buffer containing 0.01% human gamma‐globulins and incubated on ice (4°C for 15 min) to block non‐specific antibody binding. After centrifugation, the cells were incubated with fluorescence‐conjugated monoclonal antibodies specific for CD86, CD54, and mouse IgG1 (isotype control) for 30 min at 4°C. The stained cells were then washed with FACS buffer and resuspended in a propidium iodide (PI)‐containing solution to assess viability before flow cytometric analysis. The expression levels of CD86 and CD54 were quantified through flow cytometry, and the Relative Fluorescence Intensity (RFI) was calculated based on the geometric Mean Fluorescence Intensity (MFI) of treated versus control samples. The RFI of CD86 and CD54 served as a direct indicator of the test compound's potential to induce dendritic cell activation, with threshold values used to classify the substance as a sensitizer or non‐sensitizer. This assay provides a mechanistic and quantitative approach to evaluating potential skin sensitizers, offering a reliable and regulatory‐compliant method for in vitro assessment of immune system activation by chemical and biological compounds.

The assay is considered valid if the following criteria are met:

Negative control: CD86 RFI < 150, CD54 RFI < 200, and cell viability > 90%.

Positive control (NiSO4): CD86 RFI > 150, CD54 RFI > 200, and cell viability > 50%.

### 2.7 Sensitisation

The KeratinoSens [21] assay was conducted to assess the ability of ADVs to activate the Nrf2‐ARE pathway, a key molecular event in the early stages of skin sensitization. This in vitro test is based on a genetically modified HaCaT human keratinocyte cell line, which contains a luciferase reporter gene under the control of the Antioxidant Response Element (ARE) promoter. The assay was performed following standard protocols, ensuring adherence to OECD Test Guideline No. 442D for skin sensitization testing. To prepare for the assay, KeratinoSens cells were seeded into 96‐well plates at a density of 10^4^ cells per well and allowed to adhere for 24 h under standard culture conditions. After this incubation period, cells were treated with the test item ADVs, prepared as a series of serial dilutions to cover a range of concentrations. The positive control, ethylene glycol dimethacrylate (EGDMA), and the negative control, 1% dimethyl sulfoxide (DMSO), were included in parallel to ensure assay validity. The exposure period lasted 48 h, during which cells were maintained in a humidified incubator at 37°C with 5% CO_2_ to ensure optimal growth conditions.

After exposure, two primary endpoints were measured: luciferase induction, as an indicator of ARE‐dependent gene expression, and cytotoxicity, assessed to ensure that observed effects were not a consequence of cell death. Luciferase activity was quantified using the One‐Glo reagent (Promega), which provides a luminescence‐based readout of transcriptional activation. The luminescence intensity was recorded using a plate reader with an integration time of 1 s per well. To evaluate cytotoxicity, the resazurin assay was performed by adding 20 μL of resazurin solution to each well and incubating for 4 h before measuring fluorescence at 540Ex/590Em nm. The viability of the cells was calculated relative to the negative control to determine IC50 and IC30 values, which correspond to the concentrations causing 50% and 30% reductions in cell viability, respectively. Key quantitative parameters were determined to classify the test item. Imax was calculated as the maximum fold induction of luciferase activity observed at any concentration. EC1.5, the concentration at which luciferase activity exceeded 1.5‐fold relative to the control, was derived using linear interpolation. To be classified as a sensitizer, the test item had to meet at least two out of four defined criteria in two out of three independent experimental repetitions: (1) an Imax ≥ 1.5‐fold, (2) an EC1.5 value below 1000 μM, (3) cell viability above 70% at EC1.5, and (4) a dose‐dependent increase in luciferase activity. For quality control, positive control criteria were verified, including a statistically significant gene induction of at least 1.5‐fold, an average Imax between 2 and 6‐fold at 250 μM, and an EC1.5 value within 30–100 μM. The negative control was assessed for variability, which had to remain below 20% per experimental repetition for the results to be considered valid. Additionally, statistical significance was determined using a two‐tailed Student's *t*‐test (*p* < 0.05) to compare luciferase activity in test samples against the negative control. To ensure that gene induction was not an artifact of cytotoxicity, it was verified that the lowest concentration with ≥ 1.5‐fold luciferase induction maintained cell viability above 70% and remained below the IC30 threshold. Furthermore, a dose‐dependent increase in luciferase activity was considered essential for a positive classification. For multi‐constituent substances or complex mixtures, potential interference from cytotoxic components was considered, as high levels of non‐sensitizing cytotoxic constituents could mask the response of weaker sensitizers. If necessary, testing of individual components or fractionation strategies were recommended to accurately assess sensitization risk. By adhering to these standardized methods, the KeratinoSens assay provided a reliable and reproducible assessment of the test item's potential to activate keratinocyte stress response pathways involved in skin sensitization.

### 2.8 Lenitive

This study aimed to evaluate the effectiveness of cosmetic products containing soothing ingredients by assessing their impact on skin irritation. A total of 20 healthy volunteers participated in the trial, during which the test product was applied to designated areas on the volar surface of their forearm. To simulate irritation, a chemical agent was used to induce an acute inflammatory response, after which skin redness was monitored through instrumental measurements for a duration of 16 min following both the removal of the irritant and the application of the test product. In order to provide a benchmark for comparison, a commercially available formulation containing hydrocortisone acetate was included as a positive control. The collected data were analyzed using appropriate statistical techniques to ensure reliability and accuracy in the results. Participants were selected based on specific inclusion criteria, which required them to be Caucasian adults between the ages of 18 and 65, of either gender, and in good overall health, with no ongoing or pre‐existing medical conditions at the time of the study. Individuals who did not meet these requirements were excluded, as were those undergoing topical or systemic treatments with medications that could potentially interfere with the study outcomes, such as anti‐inflammatory drugs or steroids. Additional exclusion factors included pregnancy, breastfeeding, the presence of dermatological conditions in the test areas, and a history of skin disorders such as dermatitis or psoriasis. Subjects with known hypersensitivity to any of the ingredients in the tested formulation were also ineligible for participation. Participants were given the option to withdraw from the study at any point, either voluntarily or in the event of adverse reactions such as irritation or allergic responses. Before the study commenced, all individuals were fully informed about the research objectives and potential discomforts, and written consent was obtained to confirm their willingness to take part. For objective evaluation, an advanced instrument equipped with a microchip‐based probe was used to measure skin color under standardized conditions. The results were expressed as a quantitative measure of redness. This allowed for an accurate comparison between the treated and control areas, thereby assessing the test product's ability to alleviate irritation and reduce skin redness. The study was conducted in a controlled environment with a stable temperature of 21°C ± 2°C and relative humidity of approximately 50%. To induce inflammation‐mediated oxidative stress, a solution of Methyl Nicotinate, a well‐known irritant and rubefacient, was applied to randomized one‐square‐centimeter areas on the volar forearm. The irritant remained in place for 3 min before being removed, at which point the test formulation was applied in a precise amount of 50 mg ± 2 mg. Instrumental measurements were performed at multiple intervals—15, 30, and 60 min post‐application—with each recorded value representing the mean of four separate readings. To ensure the accuracy of the findings, all collected data underwent statistical analysis. Comparisons of mean values were conducted using either the Student's *t*‐test for paired samples or the Wilcoxon signed‐rank test, depending on data distribution. Statistical significance was established at a threshold of *p* ≤ 0.05, ensuring that the observed effects were not due to random variation but reflected a meaningful response to the tested formulation.

### 2.9 Skin Aging Process

The skin aging process is characterized by the progressive loss of structural components, leading to the formation of wrinkles and alterations in skin texture. This study aimed to assess the anti‐wrinkle efficacy of a cosmetic formulation containing 2% ADV‐based formulation through non‐invasive instrumental evaluation. The Antera 3D imaging system was utilized to quantify parameters related to wrinkle length, depth, volume, and skin roughness over a period of 60 days following twice‐daily application of the product. The study was conducted under controlled environmental conditions, with a temperature of 21°C ± 2°C and relative humidity of approximately 50%. Participants were acclimatized for 15 min before undergoing measurements. A total of 20 healthy volunteers, aged 41–69 years, applied the product twice daily (morning and evening) for 60 consecutive days.

### 2.10 Exclusion Criteria

Subjects were excluded if they:

Did not meet the inclusion criteria.

Were undergoing topical or systemic pharmacological treatments that could interfere with the study outcomes (e.g., corticosteroids, anti‐inflammatories).

Were pregnant or breastfeeding.

Had dermatological conditions in the test areas (e.g., dermatitis, psoriasis, hypersensitivity to product ingredients).

### 2.11 Instrumentation and Measurements

The Antera 3D system was employed to evaluate the skin's micro‐relief and wrinkle morphology. This system provides a quantitative and reproducible assessment of skin surface roughness by analyzing vertical deviations from an ideal surface. The following parameters were measured:

Wrinkle length (mm)—total length of wrinkles in the region of interest.

Average roughness, Ra (μm)—mean roughness of the skin surface.

Total wrinkle volume (mm^3^)—overall wrinkle volume in the analyzed area.

Average wrinkle depth (mm)—depth of selected representative wrinkles.

Baseline (T0) measurements were recorded before the start of treatment, followed by evaluations at 30 days (T30) and 60 days (T60). Each measurement was the mean of three readings taken from the periocular region. Volunteers were instructed to apply an appropriate amount of the test product across the entire face.

## 3 Results

### 3.1 Genotoxicity

The genotoxicity assessment of AdVs was conducted using a bacterial reverse mutation assay, which revealed no evidence of mutagenic activity. The test involved *Salmonella typhimurium* and *Escherichia coli* strains carrying mutations in the histidine and tryptophan operons, rendering them auxotrophic for these amino acids. Following exposure to AdVs, revertant colonies capable of growing in the absence of histidine or tryptophan were analyzed. Results are reported in Table 1 were The number of revertant colonies (His +/Trp +) detected in triplicate for each concentration tested (mean, standard deviation) and the value of the treated/control ratio (t/c): a ratio of t/c that exceeds 2.0 is a first indication of mutagenicity.

**TABLE 1:** AMES Test.

	STDisc *Salmonella typhimurium* TA100		STDisc *Salmonella typhimurium* TA1535		STDisc *Salmonella typhimurium* TA1535		STDisc *Salmonella typhimurium* TA1537		*Salmonella typhimurium* TA98	
Sample	Revertant colonies	Mutagenic	Revertant colonies	Mutagenic	Revertant colonies	Mutagenic	Revertant colonies	Mutagenic	Revertant colonies	Mutagenic
Blank	3 ± 1	No	4 ± 2	No	3 ± 1	No	3 ± 2	No	4 ± 2	No
Negative control	2 ± 1	No	4 ± 1	No	5 ± 3	No	5 ± 2	No	3 ± 2	No
Positive control: ICR191	874 ± 67	Yes	1023 ± 73	Yes	1011 ± 49	Yes	1002 ± 53	Yes	992 ± 58	Yes
Positive control: Sodium Azide	915 ± 34	Yes	674 ± 43	Yes	1042 ± 37	Yes	798 ± 63	Yes	854 ± 52	Yes
Treatment	3 ± 2	No	3 ± 2	No	2 ± 2	No	3 ± 2	No	3 ± 2	No
No treatment	3 ± 1	No	3 ± 1	No	3 ± 2	No	2 ± 2	No	5 ± 2	No

Table 1 show the results of the evaluation of mutagenicity of the test item on TA98 and TA1537 *Salmonella typhimurium* strains, in absence (−) and in presence (+) of metabolic activation. The number of revertant (His+ mutant) colonies is reported for each replicate, as well as their mean values (± standard deviation), for each concentration tested. Moreover, the treated/control ratio (t/c) is reported.

The results demonstrated that AdVs did not induce a significant increase in revertant colony formation compared to the negative control across all tested strains and conditions, including with and without metabolic activation (S9 mix). The absence of dose‐dependent increases in revertant colonies further supported the non‐genotoxic nature of the test item. These findings indicate that AdVs do not exhibit mutagenic properties under the experimental conditions employed, confirming their safety for further applications.

### 3.2 Toxicity for Corneal Tissue

The potential of the test item ADVs to be hazardous for the eyes was investigated through an in vitro corneal time‐to‐toxicity study, using a commercial reconstructed human corneal epithelium (RHC) model, and following the OECD 492 Guideline. The negative and positive controls gave acceptable results, and the study was accepted as valid.

**FIGURE 1:**
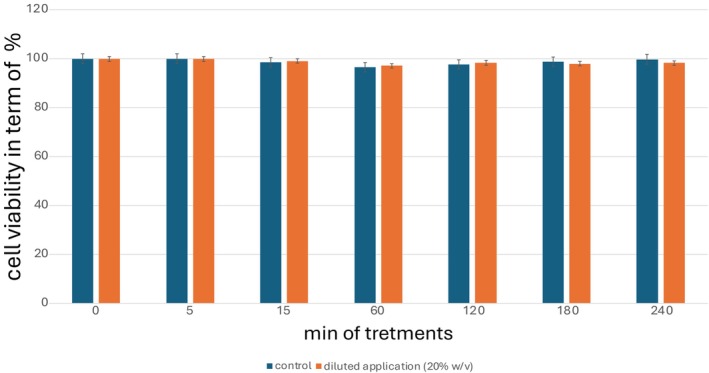
The ocular safety of ADVs was assessed using an MTT‐based in vitro corneal viability test with an RHC model, following OECD 492 guidelines. The results confirmed non‐toxicity, as no reduction in cell viability was observed.

The mean cell viability of the test item treated tissues is reported on Figure 1.

The graphical analysis demonstrates that from time 0 to 240 min of treatment, neither the untreated control tissue nor the tissue treated with apple‐derived vesicles (ADVs) exhibited a reduction in cell proliferation. This indicates that ADVs do not negatively influence cellular viability during the observation period. The absence of any significant decline in proliferation suggests that the introduction of ADVs does not trigger cytotoxic effects or alter the normal biological activity of the treated tissue. Moreover, no statistically significant differences were observed between the untreated and ADV‐treated samples, further confirming the safety profile of ADVs. This finding is particularly relevant in the context of their potential therapeutic applications, as it demonstrates that ADVs do not induce adverse effects on cell growth or metabolic activity. The stability of cellular proliferation in both groups highlights the biocompatibility of ADVs, reinforcing their potential for use in biomedical and regenerative applications. These results contribute to the growing body of evidence supporting the safe application of plant‐derived extracellular vesicles in various therapeutic and biotechnological contexts. ADVs are identified as not hazardous for the eyes and are classified as No Category according to the UN GHS classification.

### 3.3 Corrosion

The in vitro skin corrosion assay is a crucial step in evaluating the safety of a substance intended for topical or transdermal applications. This study follows the OECD 431 guideline, which is an internationally recognized method for assessing the corrosive potential of chemicals, biomaterials, and novel formulations using a reconstructed human epidermis (RhE) model. By simulating the physiological properties of human skin, this test provides a reliable and ethical alternative to traditional animal testing, allowing for an accurate assessment of potential skin damage. In this study, the positive control confirmed the expected corrosivity of the reference control, demonstrating that the assay conditions were properly established and that the test system responded appropriately to a known corrosive agent. The blank and negative controls also performed within acceptable limits, ensuring the validity and reproducibility of the results obtained for the test item (Table 2).

**TABLE 2:** Critery of Test Acceptance.

Sample	Mean	Experimental value	Limits	Results
Negative control 3 min of exposure	Mean O.D	1.98	≥ 0.8 and < 3	Compliant
Negative control 60 min of exposure	Mean O.D	1.56	≥ 0.8 and < 3	Compliant
Positive control 60 min of exposure	Mean %	7.43	≤ 15%	Compliant
Negative control 3 min of exposure	Mean (difference viability among replicates)	9.45 Max difference (% Max‐% Min)	≤ 30% In the range of 20%–100% viability or for O.D ≥ 0.3	Compliant
Negative control 60 min of exposure	Mean (difference viability among replicates)	9.45 Max difference (% Max‐% Min)	≤ 30% In the range of 20%–100% viability or for O.D ≥ 0.3	Compliant
Positive control 60 min of exposure	Mean (difference viability among replicates)	N/A Viability < 20% O.D < 0.03	In the range of 20%–100% viability or for O.D ≥ 0.3	Compliant
Test item 3 min of exposure	Mean (difference viability among replicates)	10.34 Max difference (% Max‐% Min)	≤ 30% In the range of 20%–100% viability or for O.D ≥ 0.3	Compliant
Test item 60 min of exposure	Mean (difference viability among replicates)	16.79 Max difference (% Max‐% Min)	In the range of 20%–100% viability or for O.D≥ 0.3	Compliant

**FIGURE 2:**
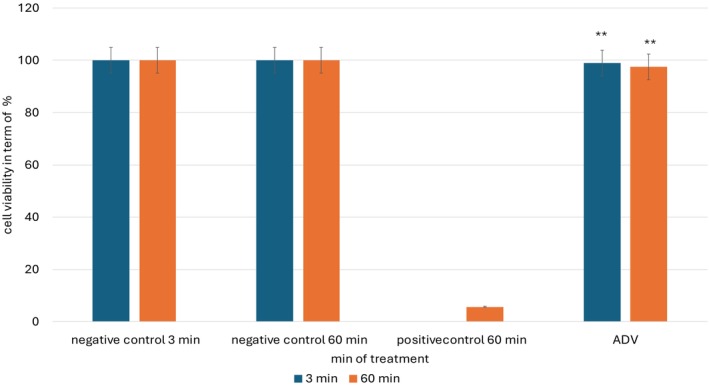
Cell viability test: Apple‐derived vesicles (ADVs) maintained 98% cell viability at 3 min and 97% at 60 min, confirming their non‐corrosive nature. As viability remained well above the 50% corrosivity threshold, ADVs are classified as biocompatible, supporting their safe use in biomedical, pharmaceutical, and cosmetic applications. ** *p* < 0.001.

This confirms that the model was functioning as intended, thereby providing confidence in the study's reliability. The test results reported on Figure 2 for the apple‐derived vesicles (ADVs) showed that the mean cell viability of treated tissues remained at 98% after 3 min of exposure and 97% after 60 min. Since a viability threshold below 50% is required to classify a substance as corrosive, these results strongly indicate that ADV does not induce significant cellular damage or compromise epidermal integrity. As a result, ADV is classified as non‐corrosive to the skin. These findings have significant implications for the biomedical, pharmaceutical, and cosmetic industries. Ensuring that a test substance does not exhibit corrosive properties is a fundamental safety requirement before advancing to further dermatological evaluations, such as irritation potential, sensitization, and long‐term compatibility studies. The high viability observed in ADV‐treated tissues suggests that these extracellular vesicles are biocompatible and suitable for applications where skin exposure is expected.

Moreover, the absence of corrosivity supports the potential use of ADV in skincare formulations, regenerative medicine, and advanced drug delivery systems, where the integrity of the skin barrier is paramount. The ability of ADVs to retain cellular viability without causing damage further strengthens their role as promising bioactive components for therapeutic and cosmetic applications. In conclusion, this study provides strong evidence of the safety profile of ADV, validating its non‐corrosive nature and paving the way for further investigations into its dermatological applications.

### 3.4 Irritation

**FIGURE 3:**
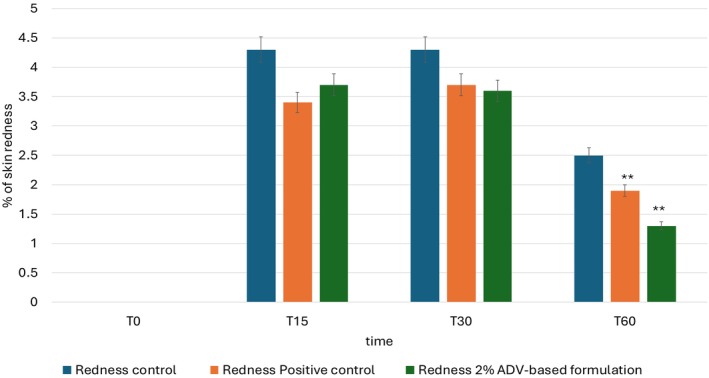
Skin irritation assessment of ADVs using an RhE model (OECD 439). After 42‐min exposure and 42‐h recovery, MTT assay confirmed no cytotoxic effects, indicating no skin irritation potential. ** *p* < 0.001.

The potential skin irritation properties of the test item AdVs were assessed using an in vitro skin irritation assay based on a reconstructed human epidermis (RhE) model. This advanced three‐dimensional skin model replicates key structural and functional characteristics of human epidermal tissue, making it a reliable system for evaluating irritation potential without the need for animal testing. The study was conducted in accordance with the OECD Test Guideline No. 439, a standardized and widely recognized protocol for determining the ability of a test substance to impair epidermal integrity and reduce cell viability, both key indicators of skin irritation. To evaluate the effects of AdVs on skin tissue, the test item was applied directly to the epidermal surface of the RhE model and incubated for 42 ± 1 min to simulate real‐world exposure conditions. Following this exposure period, the tissues underwent a 42 ± 1‐h post‐treatment recovery phase, during which cellular responses to the test item were monitored under controlled culture conditions. The assessment of skin irritation was based on the measurement of cell viability, which serves as a critical endpoint in determining whether the test item exerts cytotoxic effects. Cell viability was quantified using the MTT assay, a colorimetric technique that measures mitochondrial activity as an indicator of cell health (Figure 3). The assay is based on the enzymatic reduction of 3‐(4,5‐dimethylthiazol‐2‐yl)‐2,5‐diphenyltetrazolium bromide (MTT) into an insoluble formazan product by metabolically active cells. A higher formazan yield corresponds to greater cell viability, while a decrease in absorbance indicates potential cytotoxic effects.

According to the OECD guideline, the mean cell viability of the negative control group is considered the baseline reference value for the experiment, representing 100% viability. This reference allows for the precise comparison of treated samples and ensures that deviations in cell viability can be directly attributed to the effects of the test item. The study design and controls adhered strictly to regulatory requirements, ensuring that the data generated were both scientifically robust and compliant with international safety evaluation standards. Based on the results obtained, the test item ADVs are classified as not irritant to the skin.

### 3.5 Sensitization

**FIGURE 4:**
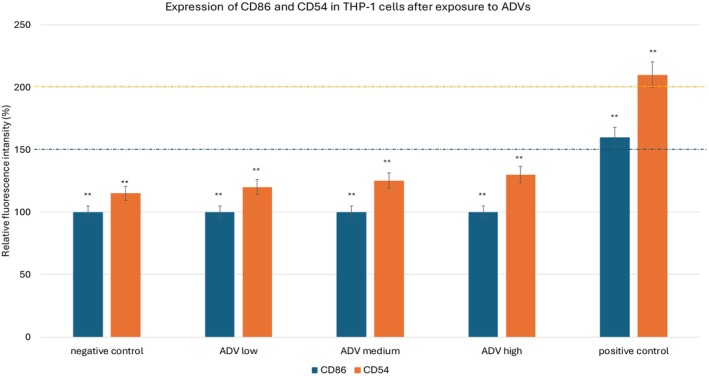
The assessment of the sensitization potential of ADVs demonstrated the absence of significant upregulation of the co‐stimulatory surface markers CD86 and CD54 in THP‐1 cells following 24 h of exposure to the test substance. This graph illustrates the results of the sensitization assay performed using THP‐1 cells. CD86 (blue) and CD54 (orange) are the two surface markers analyzed. Bars corresponding to ADV treatment at low, medium, and high concentrations show expression levels well below the regulatory thresholds (150% for CD86 blu lline, 200% for CD54 orange line). The positive control (NiSO_4_) exceeds both thresholds, confirming proper activation of the assay system. **p < 0.001.

The assessment of the sensitization potential of ADVs demonstrated the absence of significant upregulation of the co‐stimulatory surface markers CD86 and CD54 in THP‐1 cells following 24 h of exposure to the test substance. Across all tested concentrations, the expression levels of these markers remained consistently below the regulatory threshold values established for classifying a substance as a sensitizer. The study was conducted in three independent experimental runs, each performed in triplicate, ensuring the reproducibility and reliability of the findings. The negative control group, consisting of untreated THP‐1 cells, produced Relative Fluorescence Intensity (RFI) values below 150% for CD86 and below 200% for CD54, confirming the baseline conditions of the assay. This result (Figure 4) indicated that, in the absence of a sensitizing stimulus, the THP‐1 cells maintained normal expression levels of these surface molecules, as expected. In contrast, the positive control, nickel sulfate (NiSO_4_, CAS No. 10101‐97‐0), resulted in a marked increase in marker expression, generating RFI values equal to or exceeding 150% for CD86 and 200% for CD54, as required by OECD Guideline No. 442E. This confirmed the expected activation of the THP‐1 cells and validated the assay's responsiveness, ensuring that the experimental conditions were appropriate for detecting sensitizing substances. Cells treated with ADV across all tested concentrations exhibited no significant increase in CD86 or CD54 expression levels compared to the negative control. The RFI values for both markers remained well below the sensitization classification thresholds, with no evidence of a concentration‐dependent effect. Additionally, there was no indication of inter‐experimental variability, as results were consistent across all three experimental repetitions. The lack of significant upregulation of CD86 and CD54 suggests that ADV does not trigger an immune activation response in THP‐1 cells, indicating that it lacks the potential to act as a skin sensitizer. Furthermore, cell viability remained above the acceptable threshold throughout the study, confirming that the absence of marker upregulation was not due to cytotoxic effects of the test substance. This reinforces the conclusion that ADVs is non‐sensitizing under the tested conditions and does not induce an inflammatory or immune activation response. These results provide strong evidence supporting the safety profile of ADVs, particularly in applications where skin exposure is anticipated. The absence of sensitization markers confirms that ADVs does not contribute to allergic reactions or immune hypersensitivity, making it a suitable candidate for dermatological and cosmetic applications.

### 3.6 Sensitisation

**FIGURE 5:**
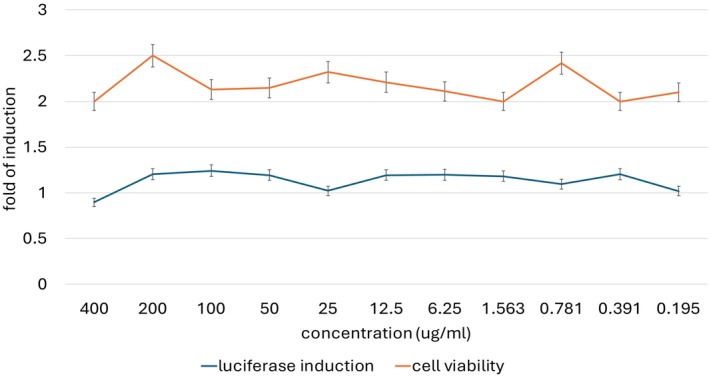
KeratinoSens assay (OECD 442D) confirmed that ADVs do not activate the Nrf2‐ARE pathway, with no sensitization potential. Luciferase expression remained below threshold, and cell viability stayed above 70%, ensuring reliability.

The assessment of the skin sensitization potential of ADVs was conducted through the KeratinoSens assay, an established in vitro method that evaluates the activation of the Nrf2‐ARE signaling pathway in human keratinocytes. This pathway is a key molecular mechanism involved in the cellular response to electrophilic and oxidative stress and represents an early event in the skin sensitization process. The assay provides a quantitative measurement of ARE‐dependent luciferase gene expression, allowing for the identification of substances capable of triggering keratinocyte activation, a crucial step in the initiation of allergic contact dermatitis. The results reported on Figure 5 of the study demonstrated that ADVs did not induce a significant increase in luciferase gene expression at any of the tested concentrations. Throughout all experimental replicates, Imax values remained consistently below the 1.5‐fold induction threshold, which is the minimal response level required for classification as a potential sensitizer according to regulatory guidelines. This finding indicates that exposure to ADVs failed to elicit a measurable transcriptional response associated with the activation of the Nrf2‐ARE pathway, suggesting that the test item does not interact with keratinocytes in a manner characteristic of sensitizing compounds. Further analysis of the dose–response relationship confirmed the absence of ARE‐mediated transcriptional activity, as no concentration‐dependent increase in luciferase expression was observed. The EC1.5 values, representing the concentration at which luciferase activity exceeds 1.5‐fold relative to the untreated control, remained above the 1000 μM threshold across all experimental conditions, further reinforcing the conclusion that ADVs lack the capacity to induce keratinocyte activation in vitro. Additionally, cell viability remained high throughout the study, with values consistently above 70% at all tested concentrations, confirming the absence of cytotoxic effects that could interfere with the interpretation of luciferase activity. The IC50 and IC30 values, which correspond to the concentrations causing 50% and 30% reductions in viability, respectively, were not reached within the tested concentration range. This indicates that ADVs exhibited no signs of cytotoxicity under the conditions employed in this study, further supporting the robustness of the findings. The validity of the assay was confirmed by the results of the control groups. The positive control, ethylene glycol dimethacrylate (EGDMA), induced a strong and reproducible increase in luciferase activity, with Imax values exceeding 1.5‐fold and EC1.5 values well below the 1000 μM threshold, demonstrating the expected activation of the Nrf2‐ARE pathway in sensitizing conditions. The negative control, 1% DMSO, produced results within the baseline range, confirming the stability and reliability of the assay system. According to the OECD 442D prediction model, a test item is classified as a sensitizer if it meets at least two of the following four criteria in two out of three independent experimental runs: an Imax of at least 1.5‐fold, an EC1.5 below 1000 μM, a cell viability of at least 70% at EC1.5, and a dose‐dependent increase in luciferase activity. In the case of ADVs, none of these criteria were met in any of the experimental repetitions, leading to its classification as non‐sensitizing.

These findings provide strong evidence that ADVs do not induce a keratinocyte‐mediated response linked to skin sensitization, indicating that the test item is unlikely to function as a skin sensitizer under the tested conditions. The absence of Nrf2‐ARE pathway activation suggests that ADVs do not act as an electrophilic or reactive substance capable of triggering an allergic skin response, supporting its non‐sensitizing nature. This conclusion enhances the safety profile of ADVs, reinforcing its suitability for applications where direct and prolonged skin contact is expected, such as in dermatological, cosmetic, and biomedical formulations.

### 3.7 Lenitive Effect

**FIGURE 6:**
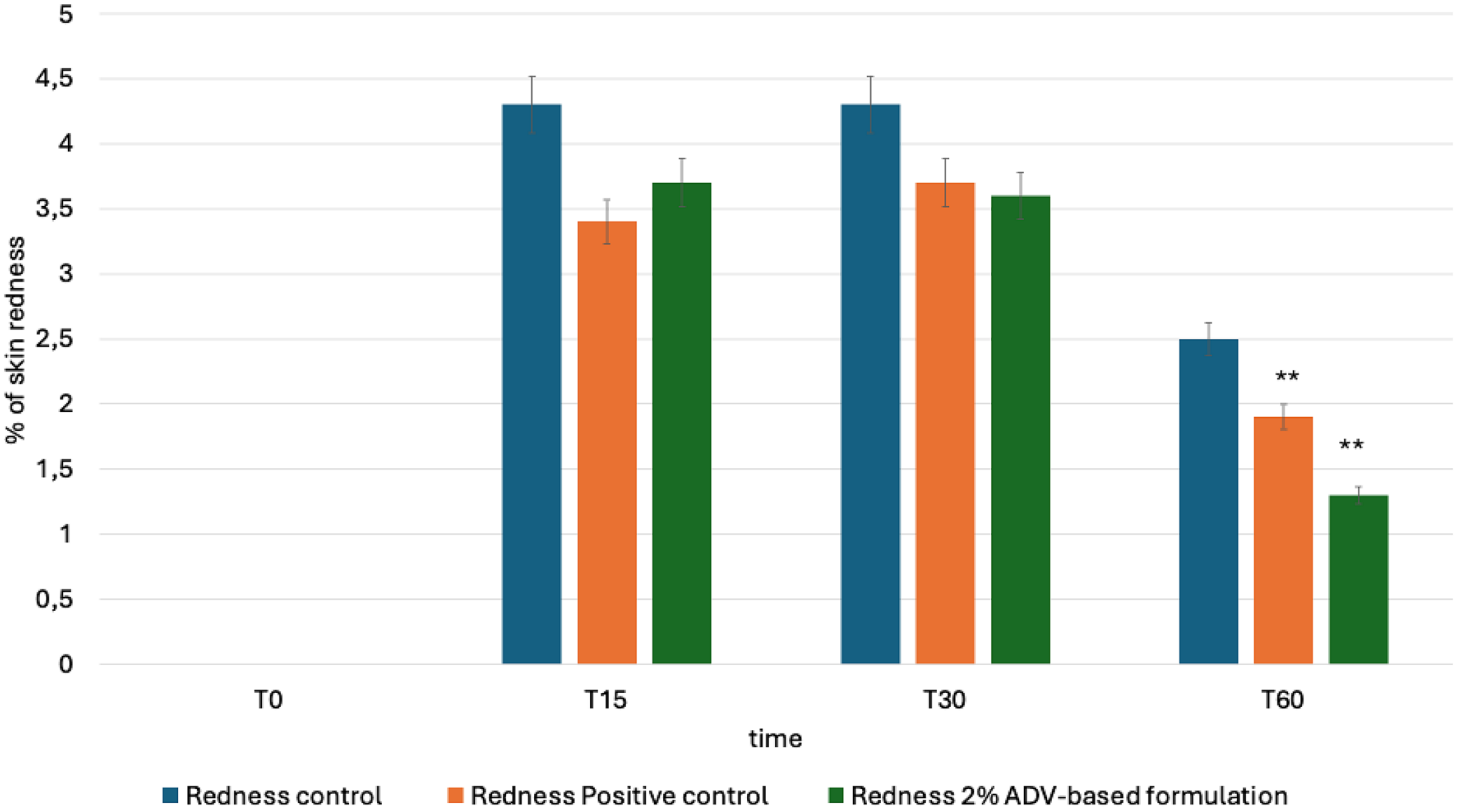
Lenitive effect: Graph reports the values for each area at different times expressed as percentage increase in skin redness compared to the basal. ** *p* < 0.001.

The results of the instrumental test related to topical treatment with 2% ADV‐based formulation are summarized in Figure 6 showing the average values for each area at different times expressed as percentage increase in skin redness compared to the basal. Based on the results of the study conducted according to the protocol described in this report, the application of ADV‐based cream 2% has been shown to be effective in reducing chemical‐induced irritation (methyl nicotinate) 60 min after its application (*p* < 0.05). The product under investigation has shown an ability to reduce skin redness through a slower, yet still effective mechanism compared to the area where the formulation containing hydrocortisone acetate, a specific drug for treating skin irritations, was applied.

The study, conducted in strict adherence to the established protocol detailed in this report, demonstrated that the application of the 2% ADV‐based formulation resulted in a statistically significant reduction in chemically induced skin irritation caused by methyl nicotinate after 60 min of treatment (*p* < 0.05). These findings suggest that the tested formulation is capable of exerting an anti‐inflammatory effect, contributing to the attenuation of skin redness over time. A comparative assessment between the test product and a hydrocortisone acetate‐containing reference formulation, a pharmacologically active compound commonly used for treating inflammatory skin conditions, revealed differences in the rate and extent of erythema reduction. The hydrocortisone‐based treatment exhibited a faster and more pronounced effect in diminishing redness, while the test product acted through a gradual yet consistent mechanism, effectively reducing erythema over a longer period. These observations highlight the potential of the 2% ADV‐based formulation as a topical treatment for skin irritation, particularly for applications where a progressive and sustained reduction in inflammation is desirable. Further studies exploring its long‐term effects, optimal dosing regimen, and potential mechanisms of action would be beneficial in elucidating its full dermatological potential.

### 3.8 Anti‐Wrinkle Effects

**FIGURE 7:**
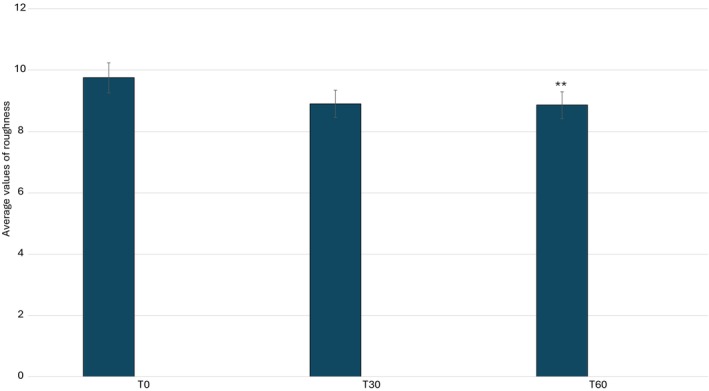
Average values of wrinkle length. **p < 0.001.

**FIGURE 8:**
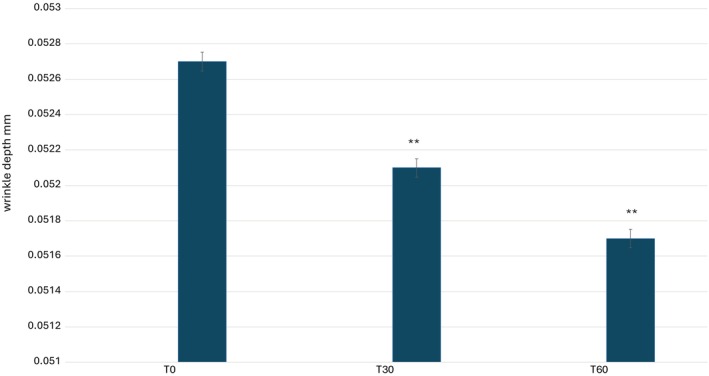
Average values of wrinkles depth. **p < 0.001.

**FIGURE 9:**
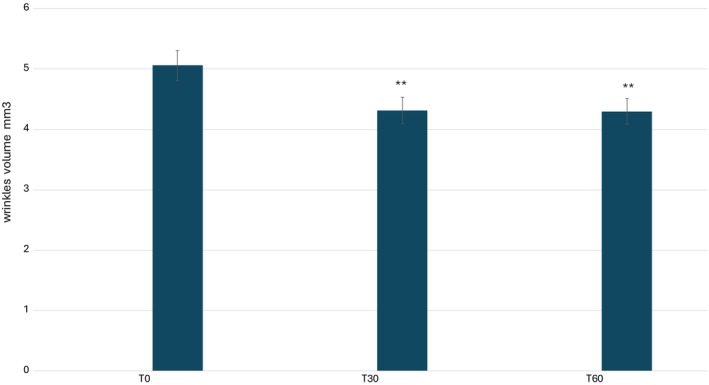
Average values of wrinkles volume. **p < 0.001.

**FIGURE 10:**
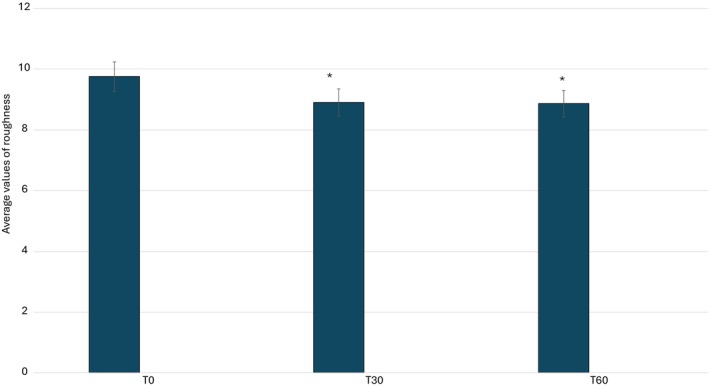
Average values of roughness. *p < 0.01.

A quantitative analysis of the study results is presented graphically in Figures 7, 8, 9, 10, illustrating the variations in key skin parameters, including wrinkle length, depth, volume, and roughness. These graphical representations provide a comprehensive overview of the measured changes, allowing for a clear comparison between baseline conditions (T0) and post‐treatment effects, further supporting the efficacy of the tested formulation. Data on wrinkle length (Figure 7), volume (Figure 8), roughness (Figure 8), and depth (Figure 10) are expressed as mean values, absolute differences, and percentage variations relative to baseline. The application of the 2% ADV‐based formulation for 60 consecutive days led to a statistically significant reduction in wrinkle length, total volume, and skin roughness in the periocular area (*p* < 0.05). These findings confirm that the formulation effectively improves skin texture and diminishes visible signs of aging. A progressive reduction in wrinkle length was observed, with a significant decrease at T60 compared to T0. Similarly, total wrinkle volume exhibited a gradual decline, reinforcing the product's ability to enhance the three‐dimensional structure of the skin. A significant improvement in skin roughness (Ra value) was also recorded, suggesting a smoother skin surface over time. However, while a slight reduction in average wrinkle depth was noted, this change did not reach statistical significance, indicating that the formulation primarily influences surface‐level skin properties rather than inducing deep tissue remodeling. Statistical analyses were performed using the Student's *t*‐test for paired samples or the Wilcoxon signed‐rank test, with a significance threshold of *p* ≤ 0.05. The results suggest that the formulation exerts a cumulative effect over time, likely attributed to the bioactive components within the formulation. While the improvement in wrinkle depth was not statistically significant, the product's impact on wrinkle length, volume, and surface texture aligns with previous research on topical treatments containing bioactive plant‐derived vesicles, known for their antioxidant and restructuring properties.

To further validate these findings, future research should investigate the long‐term effects of Melindosomes, incorporating histological analysis and biochemical markers to elucidate the biological mechanisms underlying the observed improvements. Expanding the study population and treatment duration could also provide further insights into the efficacy of the formulation across different age groups and skin types. Overall, the 2% ADV‐based formulation demonstrated significant efficacy in reducing wrinkle length, total volume, and skin roughness over a 60‐day treatment period, confirming its potential as a cosmetic intervention for improving skin appearance (Table 3). Although its impact on wrinkle depth requires further investigation, these results highlight the product's potential as an effective, non‐invasive anti‐wrinkle treatment, reinforcing its suitability for anti‐aging skincare applications.

**TABLE 3:** Effect of Wrinkle.

	Difference T30‐T0	Difference T60‐T0	% Variation T30‐T0	% Variation T60‐T0
Values of wrinkle length	5123	4817	22 674	19 877
Values of wrinkle depth	0.0005	0.0005	−1186	−1099
Values of wrinkles volume	−0.631	−0.892	15 423	13 512
Values of roughness	0.812	−0.759	−8575	−7024

## 4 Discussion

The present study offers a comprehensive evaluation of the safety profile and biological activity of apple‐derived extracellular vesicles (ADVs), highlighting their potential applicability in biomedical, dermatological, and ophthalmological contexts. The absence of genotoxic effects, as demonstrated by the Ames test, provides an essential foundation for the use of ADVs in human‐facing formulations. Across all tested bacterial strains, no increase in revertant colonies was observed, nor was there evidence of a dose‐dependent response. These findings align with regulatory expectations for non‐mutagenic substances and support the hypothesis that ADVs do not compromise genomic stability, even when administered in repeated or long‐term exposures.

In parallel, the ocular safety assessment further validated the biocompatibility of ADVs. The corneal toxicity study, which tracked cell viability over a 240‐min period, revealed no detrimental effects on corneal cells. This is particularly important given the sensitivity of ocular tissues and the growing demand for natural, non‐irritating compounds in ophthalmic product development. These data not only confirm the metabolic and structural safety of ADVs on corneal tissue but also pave the way for their use in eye care formulations, potentially replacing synthetic ingredients with lower tolerability profiles.

From a dermatological perspective, ADVs also demonstrated an excellent safety profile in vitro. Both the skin corrosion and irritation tests indicated that tissue viability remained well above the critical thresholds that define corrosive or irritant substances. These results position ADVs as favorable candidates for use in topical formulations, including products intended for individuals with sensitive or reactive skin. The lack of immune activation, as evidenced by the sensitization assays, adds another layer of confidence. No upregulation of key co‐stimulatory markers (e.g., CD86 and CD54) was observed in THP‐1 cells, and the relative fluorescence intensities remained below OECD guideline thresholds. This suggests that ADVs do not trigger hypersensitivity reactions, making them suitable for repeated dermal exposure without the risk of sensitization.

Beyond safety, the study also explored the functional bioactivity of ADVs. Notably, the anti‐inflammatory potential of a 2% ADV‐based formulation was demonstrated through its ability to mitigate methyl nicotinate‐induced erythema. Although the onset of action was more gradual compared to the hydrocortisone control, the reduction in redness was steady and sustained, pointing to a natural, biocompatible soothing effect. This observation is particularly relevant in the context of chronic inflammatory skin conditions, where long‐term corticosteroid use poses well‐known risks.

Furthermore, the anti‐aging properties of ADVs were evaluated using objective skin imaging over a 60‐day period. Improvements in surface‐level skin parameters—such as wrinkle appearance, texture, and smoothness—were statistically significant, although no major changes in wrinkle depth were detected. These data suggest that ADVs primarily influence the superficial layers of the skin, likely through hydration, barrier repair, and antioxidant activity, rather than by inducing deep dermal restructuring. This aligns with existing literature on plant‐derived extracellular vesicles, which emphasizes their role in modulating extracellular matrix dynamics, enhancing fibroblast viability, and delivering antioxidant cargo.

Collectively, these findings present a compelling case for ADVs as multifunctional bioactives with both safety and efficacy profiles that support their use in innovative cosmetic and therapeutic formulations. Importantly, the consistency across triplicate experimental runs and the reproducibility of results reinforce the robustness of the data, further validating the conclusions drawn.

## 5 Conclusion

This study establishes apple‐derived extracellular vesicles (ADVs) as safe, non‐toxic, and biologically active components with strong potential for integration into skincare, ophthalmic, and regenerative medicine applications [16, 17, 18, 19, 20]. Genotoxicity assessments confirmed the absence of mutagenic activity, while in vitro eye and skin models validated their excellent biocompatibility, showing no cytotoxic, irritant, or corrosive effects. Moreover, sensitization assays demonstrated that ADVs do not elicit immune activation or allergic responses, even at varying concentrations, confirming their suitability for formulations intended for repeated or long‐term use on sensitive skin.

Functionally, ADVs exhibited promising anti‐inflammatory effects, significantly reducing skin erythema following exposure to inflammatory stimuli. Their performance, though slower than synthetic corticosteroids, was sustained and well‐tolerated, suggesting long‐term utility in calming and repairing irritated skin. The anti‐aging efficacy of ADV‐based formulations was also substantiated, with measurable improvements in wrinkle appearance, texture, and surface uniformity, supporting their potential use in dermo‐cosmetic routines focused on skin rejuvenation.

Given their natural origin, biocompatibility, and multifunctional properties, ADVs represent a compelling alternative to synthetic actives in modern cosmetic science [21, 22, 23, 24]. Their inclusion in formulations may enhance safety, sustainability, and consumer acceptability, particularly in dermatological and ophthalmological products where tolerability and long‐term safety are critical. As extracellular vesicle technology continues to evolve, ADVs stand out as a versatile and promising platform for next‐generation skincare and regenerative therapeutics. Further studies in vivo and clinical settings will be essential to fully realize their translational potential.

## Author Contributions

L.S., M.P.C. performed the research. B.Z. designed the research study. L.F., B.Z. contributed essential reagents or tools. G.P., L.F., and B.Z. analyzed the data. B.Z. wrote the paper.

## Conflicts of Interest

The authors declare no conflicts of interest.

## Data Availability

The data that support the findings of this study are available from the corresponding author upon reasonable request.
